# Engaging men in women’s empowerment: impact of a complex gender transformative intervention on household socio-economic and health outcomes in the eastern democratic republic of the Congo using a longitudinal survey

**DOI:** 10.1186/s12889-024-17717-5

**Published:** 2024-02-12

**Authors:** Wyvine Ansima Bapolisi, Jean Makelele, Giovanfrancesco Ferrari, Lenneke Kono-Tange, Ghislain Bisimwa, Christian Schindler, Sonja Merten

**Affiliations:** 1 Université Catholique de Bukavu, Democratic Republic of the Congo, Bukavu, Sud-Kivu Democratic Republic of the Congo; 2 Swiss Tropical and Public Health Institute, Basel, Switzerland; 3 University of Basel, Basel, Switzerland; 4 CARE International, Goma, Democratic Republic of the Congo; 5 CARE Nederland, Den Haag, Nederland

**Keywords:** Microfinance, Gender, Household economy, Reproductive health

## Abstract

**Background:**

In the Democratic Republic of the Congo, women in (peri-)urban areas are commonly engaged in small trade, which allows them to meet the basic needs of their families. Microsaving approaches are a low-risk option to obtain financing for economic activities. A project combining men’s sensitization on gender equity and women’s empowerment through village savings and loan associations were implemented in North and South Kivu to raise the household economic level.

**Objective:**

This study assessed how involving men in gender equity affects women’s health and socio-economic outcomes, including food security.

**Methods:**

A cohort study was conducted with 1812 women at the baseline; out of them 1055 were retrieved at the follow-up. Baseline data collection took place from May to December 2017 and the follow-up from July 2018 to January 2019. To identify socio-economic changes and changes of gender relations, linear and logistic regressions were run.

**Results:**

Results showed that the household income improved with intervention (coefficient = 0.327; *p* = 0.002), while the capacity to pay high bills without contracting debts decreased (coefficient = 0.927; *p* = 0.001). We did not find enough statistically significant evidence of the influence of the intervention on skilled birth attendance (coefficient = 0.943; *p* = 0.135), or family planning use (coefficient = 0.216; *p* = 0.435) nor women’s participation in the decision-making (coefficient = 0.033; *p* = 0.227) nor on couple’s cohesion (coefficient = 0.024; *p* = 0.431). Food insecurity levels decreased over time regardless of being in the intervention or control area.

**Conclusion:**

Empowering women while sensitizing men on gender aspects improves financial well-being (income). Time, security, and strong politics of government recognizing and framing the approach are still needed to maximize the benefit of such projects on social factors such as women’s participation in decision-making and social cohesion.

**Supplementary Information:**

The online version contains supplementary material available at 10.1186/s12889-024-17717-5.

## Background

Programs to enhance women’s economic empowerment can spur economic growth through increased economic participation [1], which positively impacts on household welfare for example by improving household food security [2], also in humanitarian settings [3]. Positive effects were further described for the foundational drivers of household welfare that benefit the next generation, such as maternal health [4] and educational attainment [5]. Microfinance approaches are an important way of addressing women’s economic empowerment [6]. The effectiveness of microfinance programs has however shown inconsistent results for microcredit, microsavings, or microinsurance approaches [7]. Especially microcredit projects showed mixed effects, as the loans taken were hardly reimbursed– they were consumed without generating any benefits. This sometimes led to bankruptcy, worsening population impoverishment [8, 9].

A better alternative, easing the negative impact of microfinance projects, Care international and other NGOs introduced the Village Savings and Loan Associations (VSLA) approach, initially targeting primarily women [8, 10]. VSLA differs from traditional microfinance institutions in that the money for loans comes from the members themselves. The process of saving and lending money is managed by the members of the group themselves, which reduces the cost linked to microfinance institutions [8, 10]. VSLA provide poor people in remote areas with a safe place to store small amounts of cash and build up a fund from which members can take small, flexible loans [8, 11–13]. They work without long-term technical support and injections of donor capital; instead the members decide themselves about loans depending on trust, and the money stays in the community. Indeed, microsavings programs, including VSLA, have shown small but more consistent positive effects on people living in poverty, as the approach carries less risk of debt than microcredit programs [7].

In the Democratic Republic of the Congo (DRC), women in (peri-)urban areas are commonly engaged in small trade, which allows them to meet basic needs [14]. Women entrepreneurs account for more than one-third of all private sector firms, such as agriculture and informal businesses [15]. The DRC is an indebted country with the majority of the population living with less than one dollar per day [16]. Ongoing conflict since the Congo war between 1996 and 2004 displaced large parts of population in the East of the country, jeopardizing rural livelihoods as a consequence of the persistent insecurity and engendering overcrowding in towns [17]. Consequently, land was abandoned, household food production declined, and when employment became rare in rural areas, most people tried to get by through engaging in small businesses [17–19]. Women in particular have difficulty obtaining finances to develop their small-to-medium enterprises [20]. In this situation, micro-savings approaches can be a low-risk approach to improve women’s access to finance, although there may be limitations as many women cannot decide how to use their own income but have to consider their husband’s will [21]. Moreover, women’s increasing financial contributions to the household economy were not unconditionally welcomed by their husbands, despite many families being food insecure and facing problems paying medical bills or other expenses such as school fees [22–25]. The growing participation of women in the economy challenged men’s traditional breadwinner role, and some men resented that women were often not at home to pursue their household chores [14, 26]. The frustration with the changing roles and power-relations, which increases when women earn more than men, may ultimately engender intimate partner violence (IPV) [27, 28].

Concerning the determinants of household welfare, it is recognized that women’s economic empowerment can positively affect health outcomes, especially for maternal and child health [4]. This is relevant as reproductive health is foundational for the health of future generations, and given that the DRC is carrying a high burden of maternal mortality (693 per 100,000) [21]. Economic empowerment alone may however not suffice: men’s involvement in reproductive health is pivotal. A lack of participation of men in sexual and reproductive health programs and the persisting disagreement between spouses regarding their choice for the respective health service use was for example considered a main reason for the low rate of family planning (FP) uptake in the region [29, 30]. Not least from a gender equity perspective, engaging men in health programs is pivotal because women are expected to abide by their husband’s decisions before spending their own money on health care, even for their own health [21, 31–34].

### The intervention– a gender-transformative economic empowerment approach

*Mawe tatu* is a project developed by CARE in the Democratic Republic of the Congo to improve household economies and factors foundational for the health of future generations. The project combines three approaches: (i) Village Savings and Loans Associations (VSLA), as a platform that offers economic, social, and personal gains; (ii) a men-to-men sensitization to engage in learning, practice, and publicly adopt new attitudes and behaviors towards gender equality and non-violence; and (iii) education for youth to engage in healthy relationships with new models of gender equality [35].

A VSLA is a self-selected group of 15 to 30 persons who pools weekly savings of a self-defined share [10]. The savings are invested in a credit fund which is used to provide loans to members [10]. In parallel to the VSLA, women received training on topics such as gender and rights, FP or business management.

In parallel, around 30 men living in the same communities where the VSLA were introduced, are organized in reflection groups called “*baraza badirika*,” literally meaning, “peer let us change”. The men-to-men sensitization aims at developing ‘positive masculinity’ engaging men, ideally husbands/partners of VSLA’s members, towards more equitable gender norms. It is expected that men promote women’s economic empowerment and help to reduce gender-based violence. The participation in *baraza badirika* is voluntary.

This article aims to determine the impact of combining women’s empowerment through VSLA with a positive masculinity approach (“baraza badirika”) on household socio-economy (women’s income, financial resilience, household food security, women participation in decision making and cohesion), and on foundational health outcomes (reproductive health outcomes, and FP use, and gender-based violence) in a humanitarian setting in North and South Kivu Provinces, DRC.

## Methods

### Study setting

The project was implemented in eight territories of North and South Kivu: Bagira, Ibanda, Kadutu, Walungu, Goma, Karisimbi, Nyiragongo, and Rutshuru.

### Study design

A cluster-randomized intervention study was conducted for a comprehensive evaluation of the effectiveness of the *Mawe Tatu* program. Intervention and control villages were randomly selected from within the eight territories.

### Study population

In the randomly selected intervention and control villages, the village head invited women from poor households to a meeting; participation in this meeting was voluntary. In the intervention villages, meeting participants were receiving information about the VSLA approach, whereas in the control villages, an information session on an economic topic was provided. Women from villages that were randomly assigned to be in the intervention group received further introduction to the VSLA approach, while women from the control villages were only re-contacted later on for the baseline and the follow-up data collection. All women participating in these meetings who were long-term residents of their village (living in the household for at least 6 months) and were at least 15 years old were eligible for the study; based on the attendance lists, 15 women per village were randomly selected to participate in the study.

### Data collection

The baseline data collection was conducted from May to December 2017 while the follow-up took place from July 2018 to January 2019, with at least a 1-year interval (exposure) for each participant between the first (baseline) and last (endline) survey. Research assistants familiar with the local context and fluent in the local language who received 2 weeks intensive training on technical and ethical issues administered the survey questionnaires face-to-face. To ensure that participants would easily be found again, the names of the head of the household, addresses, and the mobile number of participants when available (or mobile number of someone living in the house), were obtained.

### Sample size calculation/justification

The power calculation was based on the initial hypothesis that establishing VSLA at the village level will lower the risk of stunting of children. In this article, intermediate outcomes of the program are evaluated, while the initial sample size calculation was conducted for child growth as the main outcome, which required a total sample of 800 women. Baseline data were collected in 120 selected villages (80 intervention and 40 control villages) with an average of 15 households per village. This resulted in an initial sample size of 1200 participants in 80 villages in the intervention group and 600 in the control villages. In each intervention village, 15 participants in case of one and 25 participants in case of two VSLA in the village were randomly selected from the initial meeting attendance lists. In the control villages 15 persons were randomly selected; if less participants attended the meeting, all attendees were included. After 12 months, we could re-contact 730 women in the intervention group (91.3% of the calculated sample size 800) and 316 participants in the control group (79% of the calculated sample 400) (Table 1).

Table 1 presents participants in the study at baseline and the final evaluation (follow-up).

**Table 1:** Study participants by intervention and control villages (baseline and follow-up)

	Baseline		Follow-up	
	N	%	N	%
Intervention	1225	67.6	730	69.8
Control	587	32.4	316	30.2
Total	1812	100	1046	100

Ultimately, 1046 women remained in the study at the follow-up (Table 1). The high loss to follow up (42.3% = 100*(1-(1046/1812))) can partly be explained by the fact that two villages could not be visited during the follow-up because of escalating armed conflicts in North Kivu. Therefore, all the participants of these villages were excluded. Additionally, data from one interviewer raised quality concerns and were excluded from the analysis (*n* = 79). However, the sample size calculation took into account the high rate of loss to follow-up.

A few participants (31 persons representing 3% of the final sample) switched from control to the intervention group. This concerned persons who were living in control villages but had nonetheless joined a VSLA group. Participation in VSLA was voluntary and community based i.e. people in the community were sensitized in integrating VSLA and they were called to sensitized their peers and family so that the intervention could have expanded using a snowball approach. In a VSLA program, new groups were self-initiated in addition to the initially set-up groups, and we could not prevent the ‘spill-over’ to some of the control villages. We therefore decided to re-assign all persons initially recruited in the control village who later on, nonetheless, became part of a VSLA to the intervention group.

### Instruments

The survey questionnaire includes questions about several outcomes: household economy (income, resilience, and FAO food insecurity level), maternal health services use (attendance of antenatal care, place of birth for the last pregnancy, and use of FP (modern methods), and social factors (women’s participation in decision-making, tolerance to gender based violence (GBV) against women, couple’s cohesion, and neighbor cohesion).

The same questionnaire was used for the baseline and final data collection. Some impact questions were added to the follow-up questionnaire to measure the project’s effect. For those participating in the intervention this included among others:

1. Since your husband is in a reflection group and you are in a VSLA: Can you say that your kids are going to school more regularly /Can you and your family access health care services more easily;
2. Is your husband a member of the “baraza badirika” reflection groups [if yes], since your husband is a member of the focus group/ can you easily go to the health center for ANC, post-natal care, childbirth / can you easily go to the health center for FP (information or use of a method). / Do you feel that he supports the generative activities that you do in VSLA to promote the feeding of your children? / What does your husband do for children’s nutrition?

### Measurement

Table 2 presents different variables and measurements used in this study.

**Table 2:** Measurement of main outcome variables

Variables	Type	Measurement
Household economy variables		
Income	Numeral	Income in Congolese francs or estimation (expressed in ln)
Low Resilience (resilience = ability to pay a bill of $50* without contracting a debt or asking relatives)	How would you/your household pay $50 for a hospital bill:	Using cash or savings, selling products (maize, charcoal.), selling productive assets** (goat, chickens, land) (low resilience = 0), or if had to borrow/ask money in the family, asking money from relatives, asking money from neighbors, church members, friends, making debts (low resilience = 1)
Food security	Scale (FAO)	Severe food insecurity, moderate food security, mild food insecurity, food security
Foundational health outcomes (reproductive health		
Skilled-birth attendance	Binary	Yes = 1, no = 1
ANC attendance	Binary	≥ 3 ANC during the last pregnancy = 1; <3 ANC = 0
Use of FP	Binary	Uptake of any modern FP method during the last 12 months: yes = 1 and no = 0
Gender-based outcomes		
Tolerance GBV***	Likert-scale	0–1
Neighbor/couple Cohesion	Likert-scale	0–1
Women participation in decision-making	Likert-scale	0–1

*$50 was defined after a rough estimation of the average cost for a woman to deliver without complications in a local health facility

**productive assets: definition adapted to the context [58]. One goat or three-four chickens cost approximately $50

***Tolerance to GBV against women

Household economic level was assessed using the monthly income. For those missing monthly income (Baseline *n* = 279, 40.5% Missing: *n* = 409, 59.5%; follow-up *n* = 212, 30.8%. Missing *n* = 476, 69.2%), we imputed monthly income from daily income, assets (None, radio, TV, mobile phone, electricity, computer/tablet, bicycle, motorcycle, car, boat, engine (mill, boat, other), plough, other valuables), and subjective wealth score (much poorer to much richer) as an approximation. Multiple imputation (mi) is a general approach that aims to allow for the uncertainty about the missing data by creating several different plausible imputed data sets and appropriately combining results obtained from each of them [36]. To facilitate imputation of missing data, the income variable was categorized into six classes using assets, sum and income variables; and mixed ordinal logistic regression with multiple imputations was used to analyze the ordinal variable.

Additionally, age, education, and marital status data were collected. Standard tools were used for the scales. Food insecurity was defined by the globally-employed Food and Agriculture Organization’s Food Insecurity Experience Scale (FIES) [37] used in previous studies on food insecurity [38–40]. Summative scales measuring decision-making and tolerance to gender-based violence (GBV) against women were defined according to previous studies, including the Demographic Health Surveys [21, 40, 41]. Couple and neighbor cohesion scales used were measured using the Mokken scale defined in previous studies [42]. All scales and individual item results are presented in tables as supplementary material.

### Data analysis

Descriptive statistics were calculated for socio-economic variables. A threshold of 80% was used. Participants in the VSLA group (intervention group) are coded as G = 1 as opposed to the control group (G = 0). Participation in a VSLA was investigated at the beginning of the study (T = 0) and one year after the implementation (T = 1).

For each main outcome variable, the treatment effect β is the difference between the intervention and control groups one year after the start of the program, adjusting for the difference between the two groups one year before the intervention:

1 $$\begin{array}{l}{\rm{{\rm B} = }}\,{\rm{E}}\,\left( {{\rm{Y|}}\,{\rm{G = 1,}}\,{\rm{T = 1}}} \right)\,{\rm{-}}\,{\rm{E}}\,\left( {{\rm{Y|}}\,{\rm{G = 0,}}\,{\rm{T = 1}}} \right)\,{\rm{-}}\,\\{\rm{E}}\,\left( {{\rm{Y|}}\,{\rm{G = 1,}}\,{\rm{T = 0}}} \right)\,{\rm{-}}\,{\rm{E}}\,\left( {{\rm{Y|}}\,{\rm{G = 0,}}\,{\rm{T = 0}}} \right)\end{array}$$

It was assumed that the gap between the intervention and the control groups in the following period would have been unchanged if VSLA had not been implemented.

To account for the sampling frame, multilevel mixed-effect logistic regression models were run to estimate the impact of VSLA on socio-economic factors (income-generating activities, resilience, women participation in decision-making, and cohesion) and maternal health outcomes (ANC and skilled-birth attendances and use of FP).

## Results

### Socio-demographic characteristics of participants

Table 3 presents the socio-demographic characteristics of the population in the study.

**Table 3:** Socio-demographic characteristics of participants (baseline and follow-up)

	Baseline			Follow-up		
	*Intervention*	*Control*	*Total*	*Intervention*	*Control*	*Total*
	N (%)	N (%)	N (%)		N (%)	
Province	*n* = 1239	*n* = 573	*n* = 1812	*n* = 730	*n* = 316	*n* = 1046
North-Kivu	600(48.4)	269(47.0)	869(48.0)	282(38.6)	115(36.4)	397(38.0)
South-Kivu	639(51.6)	304(53.0)	943(52.0)	448(61.4)	201(63.6)	649(62.0)
Urban	*n* = 1001	*n* = 470	*n* = 1471	*n* = 721	*n* = 314	*n* = 1035
Rural	213(21.3)	63(24.51)	317(21.6)	171(23.7)	57(18.1)	228(22.0)
Urban	631 (63.0)	169(65.76)	940(63.9)	485(67.3)	225(71.7)	710(68.6)
Semi-urban	157(15.7)	25(9.73)	214(14.5)	65(9.0)	32(10.2)	97(9.4)
Youth	*n* = 1239	*n* = 573	*n* = 1812	*n* = 730	*n* = 316	*n* = 1046
> 25	1046(84.4)	403(70.3)	1449(80.0)	665(91.1)	246(77.9)	911(87.1)
<=25	193(15.6)	170(29.7)	363(20.0)	65(8.9)	70(22.1)	135(12.9)
Education	*n* = 1235	*n* = 571	*n* = 1806	*n* = 729	*n* = 315	*n* = 1044
None	222(18.0)	123(21.5)	345(19.1)	145(19.9)	69(21.9)	214(20.5)
Primary	441(35.7)	158(27.7)	599(33.2)	234(32.1)	79(25.1)	313(30.0)
Secondary	514(41.6)	251(44.0)	765(42.4)	319(43.8)	147(46.7)	466(44.6)
Tertiary	58(4.7)	39(6.8)	97(5.4)	31(4.2)	20(6.3)	51(4.9)
Married	*n* = 1238	*n* = 572	*n* = 1810	*n* = 730	*n* = 316	*n* = 1046
No	266(21.5)	154(26.9)	420(23.2)	143(19.6)	75(23.7)	218(20.8)
Yes	972(78.5)	418(73.1)	1390(76.8)	587(80.4)	241(76.3)	828(79.2)

Compared to the intervention group, the proportion of young people (age ≤ 25 years) was higher in the control group (baseline: intervention group 15.6% (*n* = 193) vs. control group 29.7% (*n* = 170) and follow-up: intervention group 8.9% (*n* = 65) control 22.1% (*n* = 70), (Table 3)

Low resilience increased in both control and intervention groups. In both control and intervention areas, the percentage of households without food insecurity increased from 8.9% (*n* = 51) to 14.9% (*n* = 47) for the control group and 5.3% (*n* = 66) to 10.3% (*n* = 75) for the intervention group (Table 4).

**Table 4:** Distribution of the main and the secondary outcome variables

Distribution of main and secondary outcome variables						
Variables	Baseline			Follow-up		
	*Intervention*	*Control*	*Total*	*Intervention*	*Control*	*total*
Income generating activities:	*n* = 1239	*n* = 573	*n* = 1812	*n* = 730	*n* = 316	*n* = 1046
One activity ^a^	537(43.3)	305(53.2)	842(46.5)	395(54.1)	147(46.5)	542(51.8)
More than one activity ^a^	702(56.7)	268(46.8)	970(53.5)	335(45.9)	169(53.5)	504(48.2)
Daily Income (FC) ^b^	14,332 ± 41,510	9731 ± 14,929	13,387 ± 37,652	16,882 ± 36,654	10,127 ± 23,194	15,403 ± 34,247
Monthly Income (FC) ^b^	249,761 ± 2,195,756	155,208 ± 365,608	227,927 ± 1,931,057	264,411 ± 1,462,997	158,691 ± 172,270	241,733 ± 1,299,214
Low resilience:	*n* = 1239	*n* = 573	*n* = 1812	*n* = 730	*n* = 316	*n* = 1046
No ^a^	638(51.5)	255(44.5)	893(49.3)	327(44.8)	178(56.3)	505(48.3)
Yes ^a^	601(48.5)	3138(55.5)	919(50.7)	403(55.2)	138(43.7)	541(51.7)
Food insecurity:	*n* = 1239	*n* = 573	*n* = 1812	*n* = 730	*n* = 316	*n* = 1046
None ^a^	66(5.3)	51(8.9)	117(6.5)	75(10.3)	47(14.9)	122(11.7)
Mild ^a^	114(9.2)	45(7.9)	159(8.8)	59(8.1)	29(9.2)	88(8.4)
Moderate ^a^	221(17.8)	89(15.5)	310(17.1)	133(18.2)	48(15.2)	181(17.3)
Severe ^a^	838(67.7)	388(67.7)	1226(67.7)	463(63.4)	192(60.7)	655(62.6)
**Foundational health outcomes**						
Variables	Baseline			Follow-up		
	*Intervention*	*Control*	*Total*	*Intervention*	*Control*	*total*
Place of last delivery:	*n* = 1142	*n* = 482	*n* = 1624	*n* = 697	*n* = 285	*n* = 982
Skilled-birth attendance ^a^	1036 (90.7)	436 (90.5)	1472(90.6)	634(90.0)	254 (89.1)	888(90.4)
Attendance of ANC:	*n* = 1239	*n* = 573	*n* = 1812	*n* = 730	*n* = 316	*n* = 1046
< 3 ANC ^a^	132 (10.7)	71 (12.4)	203(11.2)	76(10.4)	28(8.9)	104(9.9)
≥ 3 ANC ^a^	1107(89.3)	502(87.6)	1609(88.8)	654(89.6)	288(91.1)	942(90.1)
Use of FP	*n* = 1239	*n* = 573	*n* = 1641	*n* = 730	*n* = 316	*n* = 1046
No ^a^	1133 (91.4)	508 (88.7)	1641(90.6)	654(89.6)	268(84.8)	922(88.1)
Yes ^a^	106 (8.6)	65(11.3)	171 (9.4)	76 (10.4)	48 (15.2)	124 (11.9)
**Gender-based outcomes**						
Variables	Baseline			Follow-up		
	*Intervention*	*Control*	*Total*	*Intervention*	*Control*	*total*
Women participation in decision-making^*, b^	0.69 ± 0.28	0.59 ± 0.31	0.66 ± 0.30	0.75 ± 0.28	0.67 ± 0.29	0.72 ± 0.28
Tolerance to GBV^*, b^	0.41 ± 0.21	0.41 ± 0.26	0.42 ± 0.26	0.43 ± 0.28	0.45 ± 0.29	0.44 ± 0.26
Neighbor cohesion^*, b^	0.82 ± 0.24	0.76 ± 0.26	0.80 ± 0.24	0.85 ± 0.21	0.85 ± 0.22	0.85 ± 0.22
Couple cohesion^*, b^	0.87 ± 0.28	0.82 ± 0.33	0.85 ± 0.24	0.86 ± 0.29	0.87 ± 0.28	0.86 ± 0.29

* likert scale: included items are presented in tables as supplementary material (Additional file 1)

^a^ Data variables presented as percentage of overall population n(%)

^b^ Data variables presented as Mean ± SD

Overall, the percentage of FP users increased in both groups, intervention and control from 8.6% (*n* = 106) at the baseline to 10.4% (*n* = 120) at the follow-up and from 11.3% (65) to 15.2% (48) respectively. Over the time, the percentage of skilled-birth attendance and ANC did not change in the intervention group (Table 4)

### Intervention effects on main outcomes

Table 5 presents economic outcomes by the assumed risk factors after controlling for age, education, and marital status. For each regression, only estimates of the intervention on the outcome are shown.

**Table 5:** Estimates of the impact of the intervention on socio-economic and health outcomes

Outcomes	Estimates	95% CI		*p*
Income (mi)	0.395	0.141	0.650	0.002*
FAO food insecurity	0.261	-0.363	0.887	0.412
Low resilience^**^	0.927	0.379	1.474	0.001*
Neighbor cohesion	0.007	-0.041	0.056	0.764
Couple cohesion	0.024	-0.035	0.084	0.431
Woman’s participation in decision-making	0.033	-0.021	0.088	0.227
Tolerance to GBV***	0.040	-0.016	0.097	0.159
Attendance of ANC^**^	-0.065	-0.583	0.452	0.805
FP use^**^	0.216	-0.326	0.759	0.435
Birth place^**^	0.943	-0.293	2.180	0.135

* *p* value remains statistically significant after Bonferroni and Bonferroni-Holm corrections

^**^logistic regression

*** Tolerance to GBV against women

Income was positively associated with the intervention (coefficient = 0.395; *p* = 0.002), and significantly increased low financial resilience (coefficient = 0.927; *p* = 0.001) (Table 5). The other outcomes (FP use and skilled birth and ANC attendances, tolerance to GBV against women, and intermediate outcomes like women’s participation in decision-making, neighborhood and couple cohesion) were not statistically significantly associated with the intervention. The detailed results of each outcome are presented in tables as supplementary material (Additional file 2).

## Discussion

VSLA combined with a positive masculinity program component improved the household income in this highly insecure area, while financial resilience seemed to have decreased with the intervention. We did not find significant statistical results showing the impact of the intervention on women’s participation in decision-making and cohesion or health outcomes after one year of program activities.

### Economic outcomes

We found that VSLA helped people to increase their income, corroborating what was described elsewhere [11, 43]. In the present study, the considerable number of the baseline controls shifting to the intervention group at the end of the assessment period reflects that VSLA are perceived to really help households to improve their economic status. However, the resilience (= ability to pay a bill of $50 (hospital bill for example) without contracting a debt or selling assets) declined with the intervention. This may be explained by the savings approach– the money is paid into the VSLA pool and no longer immediately accessible, which may seemingly lead to decreasing financial resilience. Even though the VSLA is considered a secure place where people can take “secure” loans instead of pledging their house or land [12, 44], being part of a VSLA is not without risk according to what was found elsewhere [28]. Women with economic hardship are more likely to have debts in VSLA since they cannot raise enough money to support their membership. Some women also belonged to several VSLA, which brought them in the situation that they could not keep up reimbursing all the loans without contracting debts elsewhere. The risk-free attitude toward making debts is also explained by the hope that they will have enough money for reimbursement after the sharing of savings at the end of a cycle. Consequently, women enter into a vicious circle of taking loans to reimburse previous ones rather than using the savings in the VSLA to improve their welfare. However, more studies are needed to understand the characteristics, purposes, and motivations of VSLA members continuing to make debts.

The surprisingly inverse relationship between the improvement in household income, on the one hand, and the reduction in the ability to pay high bills (resilience) without getting into debt, on the other, can also be partly explained by the depreciation of the national currency. At the beginning of the project, 1 US dollar was equivalent to 960 Congolese francs. One year after 1 US dollar was equivalent to 2000 Congolese francs. At the local market, the goods are appraised and sold using the fiscal franc, which in principle is equivalent to the value of US dollars. Although women can be making savings and benefits, they end up with money that is not valued in the market.

The level of food security was not affected by the program contrary to findings elsewhere [11, 44]. In the study area, many villages are facing high levels of food insecurity due to displacement and difficulty to invest in such precarious conditions [19, 45]. The short duration of the follow-up period has to be considered though, as long-term members are generally faring better than recent joiners [44].

### Maternal health and socio-gendered outcomes

We did not find statistically significant evidence of an impact of the intervention on maternal health outcomes (place of birth of the last child, attendance of antenatal care, and use of FP). In the case of this study, this can be explained by an already high percentage of attendance at health facilities for delivery (> 93%) and for antenatal care at the baseline, which means that any positive effect would be small. For FP, although there was an increase in the prevalence of those using FP at the end of the project with the intervention group, the intervention was not statistically associated with an increased in the use of FP.

In the literature, “men engage” interventions had variable effects and sometimes negative outcomes were identified [46–49]. For example, it was described that male involvement increased institutional delivery, postnatal services attendance and skilled birth attendance but did not affect birth preparedness, ANC utilization or miscarriages, and had mixed effects on breastfeeding and newborn survival [46–48]. However, in the case of our study, the short interval of time for assessing the change could also explain why no changes were found. On another note, having the majority of participants meeting the number of ANC sessions or skilled-birth attendance could explain why there were no remarkable changes because the sensitization only reinforced in what they had already been doing well.

### Gender equity, male engagement, women’s empowerment through VSLA and social change

For the social factors, the intervention did not statistically influence the women’s participation in decision-making nor the neighbor’s or the couple’s cohesion nor GBV tolerance.

The underlying theory of change in *Mawe Tatu* was that the combined introduction of VSLA for women with men’s engagement against violence towards women would improve the household’s economic level and also ultimately lead to health gains. Behavioral change is a long process that does not depend only on the availability or intention to change. Many have defined different steps that shape a change of behavior happening in a given society.

One of the theories defined six stages before a behavioral change can be observed: pre-contemplation, contemplation, preparation, action, maintenance, and termination (in a given population, 40% are in pre-contemplation, 40% in contemplation, and only 20% in preparation) [50]. However, the decision to uptake new social/health behavior is complex, specific to different contexts, and individuals. A change in behavior is more complex and there is a need to understand all the emotions of the individual and its particular context. Hanks and others, for example, showed that having the intention to use FP does not lead ultimately to the uptake of contraceptives; between intention and action, there are cultural repertoires that mediate and need to be well understood [51, 52].

The equation becomes even more complicated when gender-transformative approaches are associated with health behavior changes. Every society, mainly in SSA, has its long past of traditions and culture, and this plays an important role in how people react to social change specifically when touching sensitive topics such as gender dynamics, gender equity or reproductive health. Health promotion programs will be able to produce unprecedented impacts on the entire at-risk population if results with stage-matched interventions continue to be replicated [50].

Our results fit well with the theory of change. Indeed, some positive changes, particularly regarding the increase in income while, for other outcomes, these quantitative results (on a large sample, subject to the relatively high rate of loss to follow-up) showed very little or no change.

The fact that social changes are hardly obtained can explain the absence of any positive change on social factors in this short period of evaluation [53, 54]. Contrary to a previous qualitative study, we did not find enough evidence that engaging men besides women would be advantageous for gender equity and reducing GBV [55]. A recent review concluded that women’s economic empowerment programs challenging gender norms in a patriarchal society might generate conflicts in the couple resulting in gender-based violence [27, 56, 57]. Those conflicts may require understanding the gender dynamics to build a new balance protecting the marginalized women, which are adhered to by both men and women. It could be worthy to explore the impact of the intervention using qualitative methods to highlight the outcomes and potential conflicts generated by this gender-transformative approach. In fact, the conflicts thus generated might be counterproductive and hinder the positive effects of the intervention.

However, formative qualitative assessments in the context of the project (smaller sample but more regular follow-up) showed that some seeds are already growing; in households where the woman is a member of VSLA and the man engaged in gender equity, the socio-economic level, couple’s cohesion and dialogue within the family were improved.

Few used quantitative methods to capture the impact of the gender transformative approach associated with women’s empowerment through VSLA. This study is among the rare ones, to the best of our knowledge, trying to assess in a quantitative way the impact of a complex gender-transformative approach associated with women’s empowerment through VSLA on a combined set of outcomes (socio-economic, income, resilience, food insecurity, women’s participation in the decision-making, tolerance to GBV against women, neighbor and couple cohesion).

There is enough evidence to demonstrate that engaging men in gender equity has benefits for women [31, 55]. We do believe in accordance with Casey [31], that gender transformative approaches will require continued conceptual development of gender transformative frameworks, and particular strategies that most effectively achieve true attitudinal, behavioral, and social change.

### Limitations

During this study, a concerted effort was made to assess the impact on a wide variety of measures, while controlling for selection bias, and the results were generally encouraging. However, time, insecurity, and financial constraints severely limited both the scope and the methodological strength of the study.

Pairing VSLA members and controls was difficult in the context because there was no clear enumeration of the population. However, we did control for the sex and socio-economic backgrounds (villages in the same areas). Additionally, the percentage of women participating in VSLA with husbands/ partners as members in reflection groups was low (around 10%), which might have led to under or over-estimation of some effects of the project. Overall, 31 persons (3% of the final sample) were shifted from the control to the intervention group for this analysis because they joined the VSLA after the first round of data collection. Last, the project’s assessment period was quite short, only one year after the implementation. This short assessment period might explain why some expected results such as improved food security seems not to have been achieved. Future assessment after one or two additional years might lead to more conclusive results.

### Policy implications

- To fight poverty promote mixed programs empowering women economically through VSLA while men and women are sensitized on gender equity.
- Define adapted strategies to mitigate the easiness of taking debts can allow women to use savings for the household welfare.
- Governments and nations must provide a minimum of security to people so that they can benefit from their VSLA and invest more.

### Implications for further research

For future research projects, adjustments or additions could improve the strength of the results in several areas. First, increasing the size of the sample of those benefiting as a couple in both components of the project could improve the precision of the results. In addition, the inclusion (or redefinition) of some additional parameters may improve upon the present study. For example, some scales such as women participating in decision-making or the couple cohesion could benefit from new items in the light of qualitative findings which would improve and contextualize pre-existing scales for more accuracy.

Further research is needed to investigate more deeply the dynamics of changes happening and potential consequences such as conflicts within the couple. The low couple participation suggest that an intermediate step would be required, working with couples to get them on board for the gender specific interventions. Research is needed to define the best ways to engage more couples (husbands and wives together) in such projects.

Considering the importance of resilience, the power dynamics within the couple, and gendered parameters for both the current and future welfare of the household, it would be worthwhile to investigate these issues further. This might be achieved by using alternative parameters to measure a household’s resilience and power dynamics within the couple. Moreover, as many studies have found that female members of VSLA are more likely to contract debts, there is a need to understand how this affects the money collected, the characteristics of those contracting debts, and furthermore, a comparison between those more inclined to contract debts and those who do not. Considering the importance of income-generating activities in the field of improving household economic status, a similar study may benefit from a deeper analysis of enterprise dynamics.

## Conclusion

This analysis provides evidence that women’s empowerment through the VSLA approach associated with the male engagement in positive masculinity raises household incomes. The capacity of paying a debt without contracting any debt (resilience) decreased with the participation in VSLA. However, we did not find any effect on the household food insecurity level, women’s participation in decision-making or household cohesion. Engaging men to support women’s economic activities is of value but additional time is required to better assess the impact. The increase in income is already a premise that after years, if all the parameters are well canalized (security and strong politics to stabilize local currency), VSLA associated with men engagement towards gender equity may help to improve household socio-economic and health factors.

### Electronic supplementary material

Below is the link to the electronic supplementary material.

Additional file 1: Disaggregated scales women participation in decision-making, cohesion and tolerance GBV

Additional file 2: Detailed results of logistic and mixed regressions

## Data Availability

Dataset generated and/or analyzed are not publicly available due to confidentiality and anonymity of study population. Datasets are stored on a secure Alfresco website and are available from the corresponding author on reasonable request.

## Abbreviations

ANC: Antenatal care
DRC: Democratic Republic of the Congo
FAO: Food and Agriculture Organization
GBV: Gender based violence
FP: Family planning
VSLA: Village savings and loans associations
