# Supplementary material for: Engaging men in women’s empowerment: impact of a complex gender transformative intervention on household socio-economic and health outcomes in the eastern democratic republic of the Congo using a longitudinal survey
Source: BMC Public Health. 2024 Feb 12;24:443. doi: 10.1186/s12889-024-17717-5 (PMC10863082; doi:10.1186/s12889-024-17717-5)
Supplement: Supplementary file 1 — Additional file 1: Disaggregated scales women participation in decision-making, cohesion and tolerance GBV [file 12889_2024_17717_MOESM1_ESM.docx]

**Table S1. Women participation in decision-making**

|  |  |  | **Before** |  |  | **After** |  |  |
| --- | --- | --- | --- | --- | --- | --- | --- | --- |
|  | **Items** |  | **Intervention** | **Control** | **Total** | **Intervention** | **Control** | **Total** |
| 1 | **Who in your household usually decides on everyday expenditure?** |  | **n (%)**  **n=1227** | **n (%)**  **n=570** | **n (%)**  **n=1797** | **n (%)**  **n=727** | **n (%)**  **n=314** | **n (%)**  **n=1041** |
|  | 1 | Woman participe | 769(62.7) | 252 (44.2) | 1021(56.8) | 521(71.7) | 185(58.9) | 706(67.8) |
|  | 2 | Women does not paticipe | 458(37.3) | 318(55.8) | 776(43.2) | 206(28.3) | 129(41.1) | 335(32.2) |
| 2 | **Who in your household usually decides on special expenditures such as healtcare?** |  | **n (%)**  **n=1223** | **n (%)**  **n=569** | **n (%)**  **n=1792** | **n (%)**  **n=725** | **n (%)**  **n=314** | **n (%)**  **n=1039** |
|  | 1 | Woman participe | 657(53.7) | 232 (40.8) | 889(49.6) | 464(64.0) | 176(56.0) | 637(61.3) |
|  | 2 | Women does not paticipe | 566(46.3) | 337(59.2) | 903(50.4) | 261(36.0) | 138(44.0) | 402(38.7) |
| 3 | **More specifically, who decides where to seek care for maternal and child health issues?** |  | **n (%)**  **n=1201** | **n (%)**  **n=556** | **n (%)**  **n=1757** | **n (%)**  **n=710** | **n (%)**  **n=310** | **n (%)**  **n=1020** |
|  | 1 | Woman participe | 754(37.2) | 305 (54.9) | 1059(60.3) | 485(68.3) | 197(63.5) | 682(66.8) |
|  | 2 | Women does not paticipe | 447(44.1) | 251(45.1) | 698(39.7) | 225(31.7) | 113(36.5) | 338(33.2) |
| 4 | **Who in your**  **household usually decides if you personally want to take a**  **loan in your household ?** |  | **n (%)**  **n=1202** | **n (%)**  **n=532** | **n (%)**  **n=1734** | **n (%)**  **n=724** | **n (%)**  **n=308** | **n (%)**  **n=1032** |
|  | 1 | Woman participe | 900(74.9) | 334 (62.8) | 1234(71.2) | 583(80.5) | 218(70.8) | 801(77.6) |
|  | 2 | Women does not paticipe | 302(25.1) | 198(37.2) | 500(28.8) | 141(19.5) | 90(29.2) | 231(22.4) |
| 5 | **Who in your household decides if someone in your**  **household wants to buy or sell a mobile phone?** |  | **n (%)**  **n=1177** | **n (%)**  **n=542** | **n (%)**  **n=1719** | **n (%)**  **n=699** | **n (%)**  **n=305** | **n (%)**  **n=1004** |
|  | 1 | Woman participe | 790(67.1) | 302(55.7) | 1092(63.5) | 489(70.0) | 189(62.8) | 678(67.5) |
|  | 2 | Women does not paticipe | 387(32.9) | 240(44.3) | 627(36.5) | 210(30.0) | 116(38.0) | 326(32.5) |
| 6 | **Who in**  **your household decides regarding buying or selling land?** |  | **n (%)**  **n=1150** | **n (%)**  **n=527** | **n (%)**  **n=1677** | **n (%)**  **n=705** | **n (%)**  **n=301** | **n (%)**  **n=1006** |
|  | 1 | Woman participe | 784(68.2) | 313 (59.4) | 1097(65.4) | 529(7509) | 201(66.8) | 730(72.6) |
|  | 2 | Women does not paticipe | 366(31.8) | 214(40.6) | 580(34.6) | 176(25.0) | 100(31.8) | 276(27.4) |
| 7 | **Who in your household decides how your own income is used?** |  | **n (%)**  **n=1221** | **n (%)**  **n=559** | **n (%)**  **n=1780** | **n (%)**  **n=721** | **n (%)**  **n=312** | **n (%)**  **n=1033** |
|  | 1 | Woman participe | 1005(82.3) | 450 (80.5) | 1455(81.7) | 627(87.0) | 257(82.4) | 884(85.6) |
|  | 2 | Women does not paticipe | 216(17.7) | 109(19.5) | 325(18.3) | 94(13.0) | 55(17.6) | 149(14.4) |
| 8 | **Who in your couple decides when to have children?** |  | **n (%)**  **n=957** | **n (%)**  **n=433** | **n (%)**  **n=1390** | **n (%)**  **n=599** | **n (%)**  **n=248** | **n (%)**  **n=847** |
|  | 1 | Woman participe | 744(77.7) | 308 (71.1) | 1052(75.7) | 479(80.0) | 190(76.6) | 669(79.0) |
|  | 2 | Women does not paticipe | 213(22.3) | 125(28.9) | 338(24.3) | 120(20.0) | 58(23.4) | 213(21.0) |
| 9 | **Who in your couple decides whether to use family**  **planning?** |  | **n (%)**  **n=1027** | **n (%)**  **n=496** | **n (%)**  **n=1523** | **n (%)**  **n=624** | **n (%)**  **n=268** | **n (%)**  **n=892** |
|  | 1 | Woman participe | 841 (81.9) | 374 (75.4) | 1215(79.8) | 524(84.0) | 219(81.7) | 242(83.3) |
|  | 2 | Women does not paticipe | 186 (18.1) | 122 (24.6) | 308 (20.2) | 100(26.0) | 49(18.3) | 75(16.7) |

**Table S2. Cohesion**

|  |  |  | **Before** |  |  | **After** |  |  |
| --- | --- | --- | --- | --- | --- | --- | --- | --- |
|  | **Items** |  | **Intervention** | **Control** | **Total** | **Intervention** | **Control** | **Total** |
| 1 | **In times of crisis i can turn to my spouse/partner for support** |  | **n (%)**  **n=969** | **n (%)**  **n=425** | **n (%)**  **n=1394** | **n (%)**  **n=583** | **n (%)**  **n=249** | **n (%)**  **n=832** |
|  |  | yes | 841(86.8) | 353(83.1) | 1194(85.7) | 505(86.6) | 216(86.7) | 721(86.7) |
|  |  | no | 128(13.2) | 72(16.9) | 200(14.3) | 78(13.4) | 33(13.3) | 111(13.3) |
| 2 | **My spouse/partner and i get along well together** |  | **n (%)**  **n=956** | **n (%)**  **n=424** | **n (%)**  **n=1380** | **n (%)**  **n=572** | **n (%)**  **n=249** | **n (%)**  **n=821** |
|  |  | Yes | 868(90.8) | 370(87.3) | 1238(89.7) | 524(91.4) | 224(90.0) | 748(91.1) |
|  |  | No | 88(9.2) | 54(12.7) | 142(10.3) | 48(8.4) | 25(10.0) | 73(8.9) |
| 3 | **If i come home late without the permission of my partner/husband he gets very upset** |  | **n (%)**  **n=952** | **n (%)**  **n=412** | **n (%)**  **n=1364** | **n (%)**  **n=564** | **n (%)**  **n=247** | **n (%)**  **n=811** |
|  |  | Yes | 518(54.4) | 236(57.3) | 754(55.3) | 327(58.0) | 145(58.7) | 472(58.2) |
|  |  | No | 434(45.6) | 176(42.7) | 610(44.7) | 237(42.0) | 102(41.3) | 339(41.8) |
| 4 | **In general i can trust my neighbours** |  | **n (%)**  **n=1189** | **n (%)**  **n=531** | **n (%)**  **n=1720** | **n (%)**  **n=711** | **n (%)**  **n=300** | **n (%)**  **n=1011** |
|  |  | Yes | 830(69.8) | 343(64.6) | 1173(68.2) | 563(79.2) | 229(76.3) | 792(78.3) |
|  |  | No | 359(30.2) | 188(35.4) | 547(31.8) | 148(20.8) | 71(23.7) | 219(21.7) |
| 5 | **If my child had an accident my neighbours would immediately help** |  | **n (%)**  **n=1144** | **n (%)**  **n=492** | **n (%)**  **n=1636** | **n (%)**  **n=689** | **n (%)**  **n=286** | **n (%)**  **n=975** |
|  |  | Yes | 1051(91.9) | 427(86.8) | 1478(90.3) | 657(95.4) | 264(92.3) | 921(94.5) |
|  |  | no | 93(8.1) | 65(13.2) | 158(9.7) | 32(4.6) | 22(7.7) | 54(5.5) |
| 6 | **I often talk to my neighbours** |  | **n (%)**  **n=1227** | **n (%)**  **n=566** | **n (%)**  **n=1793** | **n (%)**  **n=723** | **n (%)**  **n=313** | **n (%)**  **n=1036** |
|  |  | Yes | 1172(95.5) | 519(91.7) | 1691(94.3) | 697(96.4) | 294(93.9) | 991(95.7) |
|  |  | No | 55(4.5) | 47(8.3) | 102(5.7) | 26(3.6) | 19(6.1) | 45(4.3) |
| 7 | **The majority of people in this community generally get along with each other.** |  | **n (%)**  **n=1137** | **n (%)**  **n=495** | **n (%)**  **n=1632** | **n (%)**  **n=670** | **n (%)**  **n=287** | **n (%)**  **n=957** |
|  |  | Yes | 933(82.1) | 384(77.6) | 1317(80.7) | 577(86.1) | 244(85.0) | 821(85.8) |
|  |  | No | 204(17.9) | 111(22.4) | 315(19.3) | 93(13.9) | 43(15.0) | 136(14.2) |
| 8 | **I feel that i am really a part of this community.** |  | **n (%)**  **n=1206** | **n (%)**  **n=546** | **n (%)**  **n=1752** | **n (%)**  **n=713** | **n (%)**  **n=300** | **n (%)**  **n=1013** |
|  |  | Yes | 1114(92.4) | 483(88.5) | 1597(91.2) | 659(92.4) | 271(90.3) | 930(91.8) |
|  |  | No | 92(7.6) | 63(11.5) | 155(8.8) | 54(7.6) | 29(9.7) | 83(8.2) |
| 9 | **I can rely on people in my community if i need to borrow money.** |  | **n (%)**  **n=1159** | **n (%)**  **n=523** | **n (%)**  **n=1682** | **n (%)**  **n=696** | **n (%)**  **n=287** | **n (%)**  **n=983** |
|  |  | Yes | 761(65.7) | 265(50.7) | 1026(61.0) | 485(69.7) | 197(68.6) | 682(69.4) |
|  |  | no | 398(34.3) | 258(49.3) | 656(39.0) | 211(30.3) | 90(31.4) | 301(30.6) |

**Table S3.** **Tolerance to SGBV**

|  |  |  | **Before** |  |  | **After** |  |  |
| --- | --- | --- | --- | --- | --- | --- | --- | --- |
|  | **Items** |  | **Intervention** | **Control** | **Total** | **Intervention** | **Control** | **Total** |
| 1 | **It is normal that a man beats his wife if she is unfaithful** |  | **n (%)**  **n=1180** | **n (%)**  **n=526** | **n (%)**  **n=1706** | **n (%)**  **n=689** | **n (%)**  **n=300** | **n (%)**  **n=989** |
|  | 1 | Yes | 733(62.1) | 318 (60.5) | 1051(61.6) | 428(62.1) | 167(55.7) | 595(60.2) |
|  | 2 | No | 447(37.9) | 208(39.5) | 655(38.4) | 261(37.9) | 133(44.3) | 394(39.8) |
| 2 | **It is normal if a parent beats a girl if she is getting pregnant outside of marriage** |  | **n (%)**  **n=1186** | **n (%)**  **n=542** | **n (%)**  **n=1728** | **n (%)**  **n=701** | **n (%)**  **n=307** | **n (%)**  **n=1008** |
|  | 1 | Yes | 522(44.0) | 208 (38.4) | 730(42.2) | 283(40.4) | 116(37.8) | 399(39.6) |
|  | 2 | No | 664(56.0) | 334(61.6) | 998(57.8) | 418(59.6) | 191(62.2) | 609(60.4) |
| 3 | **It is normal if a man beats his wife if she doesn't want to have sex with him** |  | **n (%)**  **n=1147** | **n (%)**  **n=530** | **n (%)**  **n=1677** | **n (%)**  **n=687** | **n (%)**  **n=299** | **n (%)**  **n=986** |
|  | 1 | yes | 437(38.1) | 205 (38.7) | 642(38.3) | 259(37.7) | 96(32.1) | 355(36.0) |
|  | 2 | no | 710(61.9) | 325(61.3) | 1035(61.7) | 428(62.3) | 203(67.8) | 631(64.0) |
| 4 | **If a person wastes money it is normal that he/she is beaten** |  | **n (%)**  **n=1192** | **n (%)**  **n=524** | **n (%)**  **n=1716** | **n (%)**  **n=687** | **n (%)**  **n=298** | **n (%)**  **n=985** |
|  | 1 | yes | 344(28.9) | 144 (27.5) | 488(28.4) | 204(29.7) | 70(23.5) | 274(27.8) |
|  | 2 | No | 848(71.1) | 380(72.5) | 1228(71.6) | 483(70.3) | 228(76.5) | 711(72.2) |
| 5 | **It is normal that a man beats his wife if she goes out without telling him** |  | **n (%)**  **n=1193** | **n (%)**  **n=541** | **n (%)**  **n=1734** | **n (%)**  **n=701** | **n (%)**  **n=303** | **n (%)**  **n=1004** |
|  | 1 | Yes | 373(31.3) | 186(34.4) | 559(32.2) | 245(35.0) | 101(33.3) | 346(34.5) |
|  | 2 | No | 820(68.7) | 355(65.6) | 1175(67.8) | 456(65.0) | 202(66.7) | 658(65.5) |
| 6 | **It is normal that a man beats his wife if she argues with him** |  | **n (%)**  **n=1197** | **n (%)**  **n=527** | **n (%)**  **n=1734** | **n (%)**  **n=704** | **n (%)**  **n=305** | **n (%)**  **n=1009** |
|  | 1 | yes | 339(28.3) | 192 (35.8) | 531(30.6) | 265(37.6) | 108(35.4) | 373(40.0) |
|  | 2 | No | 858(71.7) | 345(64.2) | 1203(69.4) | 439(62.4) | 197(64.6) | 636(63.0) |
| 7 | **It is normal that a man beats his wife if she neglects the children** |  | **n (%)**  **n=1206** | **n (%)**  **n=535** | **n (%)**  **n=1741** | **n (%)**  **n=697** | **n (%)**  **n=307** | **n (%)**  **n=1004** |
|  | 1 | Yes | 407(33.8) | 193 (36.1) | 600(34.5) | 627(87.0) | 257(82.4) | 399(39.7) |
|  | 2 | No | 799(66.2) | 342(63.9) | 1141(65.5) | 94(13.0) | 55(17.6) | 605(60.3) |
| 8 | **It is normal that a man beats his wife if she burns the food** |  | **n (%)**  **n=1211** | **n (%)**  **n=554** | **n (%)**  **n=1765** | **n (%)**  **n=709** | **n (%)**  **n=310** | **n (%)**  **n=1019** |
|  | 1 | yes | 140(77.7) | 63 (11.4) | 203(11.5) | 79(11.1) | 32(10.3) | 111(10.9) |
|  | 2 | No | 1071(22.3) | 491(88.6) | 1562(88.5) | 630(88.9) | 278(89.7) | 908(89.1) |
