# Supplementary material for: Engaging men in women’s empowerment: impact of a complex gender transformative intervention on household socio-economic and health outcomes in the eastern democratic republic of the Congo using a longitudinal survey
Source: BMC Public Health. 2024 Feb 12;24:443. doi: 10.1186/s12889-024-17717-5 (PMC10863082; doi:10.1186/s12889-024-17717-5)
Supplement: Supplementary file 2 — Additional file 2: Detailed results of logistic and mixed regressions [file 12889_2024_17717_MOESM2_ESM.docx]

**Detailled results of melogit and mixed regressions**

**Table S4. Income**

| **Income (MI)** | **coeff** | **95%** |  | **p** |
| --- | --- | --- | --- | --- |
| Age | 0.014 | 0.004 | 0.025 | 0.005 |
| Education |  |  |  |  |
| Primary | 0.018 | -0.427 | 0.465 | 0.934 |
| Secondary | 0.232 | -0.200 | 0.665 | 0.292 |
| Tertiary | 0.901 | 0273 | 1.530 | 0.005 |
| married | 0.607 | 0.300 | 0.913 | 0.000 |
|  |  |  |  |  |
| urban | 0.225 | -0.093 | 0.544 | 0.166 |
| Semi-urban | -0.164 | -0.721 | 0.391 | 0.561 |
| province |  |  |  |  |
| South-Kivu | -0.254 | -0.524 | 0.014 | 0.064 |
| VSLA | 0.395 | 0.141 | 0.650 | 0.002 |
| Income0 | 0.226 | 0.135 | 0.317 | 0.000 |

**Table S5. Low resilience**

| **Low resilience** | **coeff** | **95%** |  | **p** |
| --- | --- | --- | --- | --- |
| Age | -0.009 | -0.029 | 0.010 | 0.342 |
| Education |  |  |  |  |
| Primary | -0.358 | -1.239 | 0.523 | 0.426 |
| Secondary | -0.947 | -1.803 | -0.091 | 0.030 |
| Tertiary | -1.29 | -2.523 | -0.068 | 0.038 |
| Married | 0.322 | -0.264 | 0.909 | 0.281 |
|  |  |  |  |  |
| Urban | -0.152 | -0.837 | 0.531 | 0.661 |
| Semi-urban | -0.106 | -1.282 | 1.069 | 0.859 |
| Province |  |  |  |  |
| South-Kivu | 0.097 | -0.476 | 0.672 | 0.739 |
| VSLA | 0.927 | 0.379 | 1.474 | 0.001 |
| Low resilience0 | 1.05 | 0.598 | 1.516 | 0.000 |

**Table S6. FAO food**

| **FAO food insecurity level** | **coeff** | **95%** |  | **p** |
| --- | --- | --- | --- | --- |
| Age | 0.001 | -0.025 | 0.027 | 0.937 |
| Education |  |  |  |  |
| Primary | -0.782 | -2.160 | 0.595 | 0.266 |
| Secondary | -0.795 | -2.125 | 0.534 | 0.241 |
| Tertiare | -0.596 | -2.290 | 1.098 | 0.490 |
| Married | 0.751 | 0.084 | 1.417 | 0.027 |
|  |  |  |  |  |
| Urban | 0.672 | -0.083 | 1.428 | 0.081 |
| Semi-urban | 1.142 | -0.528 | 2.812 | 0.180 |
| Province |  |  |  |  |
| South-Kivu | -0.004 | -0.650 | 0.641 | 0.989 |
| VSLA | 0.261 | -0.363 | 0.887 | 0.412 |
| FAO food insecurity level/baseline |  |  |  |  |
| 1 | 0.839 | -0.224 | 1.904 | 0.122 |
| 2 | 1.610 | 0.591 | 2.628 | 0.002 |
| 3 | 2.339 | 1.369 | 3.309 | 0.000 |

**Table S7. Women participation in decision-making**

| **Women participation in decision-making** | **coeff** | **95%** |  | **p** |
| --- | --- | --- | --- | --- |
| Age | 0.004 | 0.002 | 0.006 | 0.000 |
| Education |  |  |  |  |
| Primary | -0.027 | -0.124 | 0.069 | 0.582 |
| Secondary | -0.010 | -0.105 | 0.084 | 0.827 |
| Tertiary | 0.062 | -0.068 | 0.194 | 0.348 |
| Married | 0.064 | 0.0009 | 0.128 | 0.047 |
|  |  |  |  |  |
| Urban | 0.030 | -0.036 | 0.097 | 0.374 |
| Semi-urban | 0.022 | -0.094 | 0.139 | 0.709 |
| Province |  |  |  |  |
| South-Kivu | 0.041 | -0.015 | 0.098 | 0.153 |
| VSLA | 0.033 | -0.021 | 0.088 | 0.227 |
| Womendecides0 | 0.299 | 0.207 | 0.391 | 0.000 |

**Table S8. Neighbor cohesion**

| **Neighbor cohesion** | **Coeff** | **95%** |  | **p** |
| --- | --- | --- | --- | --- |
| Age | 0.0008 | -0.001 | 0.002 | 0.406 |
| Education |  |  |  |  |
| Primary | 0.018 | -0.067 | 0.103 | 0.679 |
| Secondary | 0.016 | -0.066 | 0.099 | 0.701 |
| Tertiare | 0.019 | -0.097 | 0.136 | 0.742 |
| Married | -0.011 | -0.067 | 0.045 | 0.704 |
|  |  |  |  |  |
| Urban | -0.053 | -0.115 | 0.007 | 0.085 |
| Semi-urban | -0.080 | -0.187 | 0.026 | 0.141 |
| Province |  |  |  |  |
| South-Kivu | -0.017 | -0.070 | 0.034 | 0.504 |
| VSLA | 0.007 | -0.041 | 0.056 | 0.764 |
| Neighbor cohesion0 | 0.276 | 0.182 | 0.370 | 0.000 |

**Table S9. Couple cohesion**

| **Couple cohesion** | **Coeff** | **95%** |  | **p** |
| --- | --- | --- | --- | --- |
| Age | -0.003 | -0.006 | -0.001 | 0.001 |
| Education |  |  |  |  |
| Primary | 0.092 | -0.011 | 0.197 | 0.083 |
| Secondary | 0.113 | 0.011 | 0.215 | 0.028 |
| Tertiary | 0.131 | -0.009 | 0.273 | 0.067 |
| Married | 0.209 | 0.135 | 0.283 | 0.000 |
|  |  |  |  |  |
| Urban | 0.001 | -0.074 | 0.078 | 0.961 |
| Semi-urban | -0.149 | -0.281 | -0.017 | 0.026 |
| Province |  |  |  |  |
| South-Kivu | 0.008 | -0.054 | 0.072 | 0.787 |
| VSLA | 0.024 | -0.035 | 0.084 | 0.431 |
| Couple cohesion0 | 0.144 | 0.036 | 0.252 | 0.009 |

**Table S10. Violence scale**

| **Couple cohesion** | **coeff** | **95%** |  | **p** |
| --- | --- | --- | --- | --- |
| Age | 0.001 | -0.0008 | 0.003 | 0.210 |
| Education |  |  |  |  |
| Primary | -0.004 | -0.104 | 0.096 | 0.938 |
| Secondary | -0.086 | -0.183 | 0.011 | 0.084 |
| Tertiary | **-0.167** | **-0.305** | **-0.029** | **0.017** |
| Married | 0.029 | -0.036 | 0.095 | 0.377 |
|  |  |  |  |  |
| Urban | **-0.091** | **-0.160** | **-0.022** | **0.009** |
| Semi-urban | -0.075 | -0.197 | 0.046 | 0.226 |
| Province |  |  |  |  |
| South-Kivu | **0.062** | **0.002** | **0.122** | **0.042** |
| VSLA | 0.040 | -0.016 | 0.097 | 0.159 |
| Gemviolscale0 | **0.172** | **0.071** | **0.272** | **0.001** |

**Table S11. ANC**

| **ANC** | **coeff** | **95%** |  | **p** |
| --- | --- | --- | --- | --- |
| Age | 0.011 | -0.010 | 0.033 | 0.316 |
| Education |  |  |  |  |
| Primary | -0.293 | -1.123 | 0.536 | 0.489 |
| Secondary | 0.384 | -0.427 | 1.197 | 0.353 |
| Tertiary | 0.518 | -0.782 | 1.818 | 0.435 |
| Married | -0.005 | -0.738 | 0.727 | 0.998 |
|  |  |  |  |  |
| Urban | 0.064 | -0.576 | 0.704 | 0.844 |
| Semi-urban | 0.242 | -0.877 | 1.363 | 0.671 |
| Province |  |  |  |  |
| South-Kivu | **-0.251** | **-0.798** | **0.296** | **0.369** |
| VSLA | -0.065 | -0.583 | 0.452 | 0.805 |
| ANC0 | **1.301** | **0.834** | **1.768** | **<0.001** |

**Table S12. Skilled-birth attendance**

| **Couple cohesion** | **coeff** | **95%** |  | **p** |
| --- | --- | --- | --- | --- |
| Age | -0.032 | -0.076 | 0.011 | 0.146 |
| Education |  |  |  |  |
| Primary | -1.301 | -3.224 | 0.621 | 0.185 |
| Secondary | -0.107 | -2.098 | 1.882 | 0.915 |
| Married | 0.133 | -1.374 | 1.641 | 0.862 |
|  |  |  |  |  |
| Urban | **1.385** | **-0.0001** | **-2.770** | **0.050** |
| Semi-urban | -0.315 | -2.809 | 2.1776 | 0.226 |
| Province |  |  |  |  |
| South-Kivu | **-0.966** | **-2.644** | **0.711** | **0.259** |
| VSLA | 0.943 | -0.293 | 2.180 | 0.135 |
| Skill-birth0 | **4.041** | **2.434** | **5.648** | **<0.001** |

**Table S13. Use of FP**

| **Use of FP** | **coeff** | **95%** |  | **p** |
| --- | --- | --- | --- | --- |
| Age | -0.036 | -0.069 | -0.004 | 0.026 |
| Education |  |  |  |  |
| Primary | -0.237 | -1.367 | 0.892 | 0.681 |
| Secondary | -0.371 | -1.453 | 0.710 | 0.501 |
| Tertiary | **0.859** | **-0.476** | **2.195** | **0.207** |
| Married | **1.140** | **0.184** | **2.096** | **0.019** |
|  |  |  |  |  |
| Urban | 0.234 | -0.610 | -1.078 | 0.587 |
| Semi-urban | 0.398 | -0.969 | 1.765 | 0.568 |
| Province |  |  |  |  |
| South-Kivu | 0.113 | -0.554 | 0.782 | 0.738 |
| VSLA | -0.227 | -0.835 | 0.379 | 0.462 |
| UseFP0 | **1.062** | **0.343** | **1.781** | **0.004** |
